# Topical Instillation of N-Acetylcysteine and N-Acetylcysteine Amide Impedes Age-Related Lens Opacity in Mice

**DOI:** 10.3390/biom15030442

**Published:** 2025-03-19

**Authors:** Hidetoshi Ishida, Yu Sasaki, Teppei Shibata, Hiroshi Sasaki, Bhavana Chhunchha, Dhirendra P. Singh, Eri Kubo

**Affiliations:** 1 Department of Ophthalmology, Kanazawa Medical University, Kanazawa 9200293, Japan; ishi1214@kanazawa-med.ac.jp (H.I.); sasaki-y@kanazawa-med.ac.jp (Y.S.); prefse74@kanazawa-med.ac.jp (T.S.); mogu@kanazawa-med.ac.jp (H.S.); 2 Department of Ophthalmology and Visual Sciences, University of Nebraska Medical Center, Omaha, NE 68198, USA; bchhunchha@unmc.edu (B.C.); dpsingh@unmc.edu (D.P.S.)

**Keywords:** cataract, oxidative stress, N-acetylcysteine amide, TXNIP, NLRP3 inflammasome, lens opacity, antioxidant therapy

## Abstract

Cataracts, the leading cause of blindness globally, are caused by oxidative stress and inflammation, which disrupt lens transparency due to increased accumulation of reactive oxygen species (ROS) as well as protein and DNA damage during aging. Using in vitro, ex vivo, and in vivo models, we determined the protective efficacy of N-acetylcysteine amide (NACA) against oxidative stress-induced and aging-induced cataractogenesis. We found that lens epithelial cells exposed to the oxidative stress inducers hydrogen peroxide (H_2_O_2_) or tert-butyl hydroperoxide showed significant ROS accumulation and reduced cellular viability. These effects were inhibited by NACA via the suppression of ROS and thioredoxin-interacting protein (*Txnip*) expression, a regulator of oxidative stress-related cellular damage and inflammation. In ex vivo lens experiments, NACA significantly reduced H_2_O_2_-induced lens opacity and preserved lens integrity. Similarly to NACA-treated lenses ex vivo, the integrity and opacity of aged mouse lenses, when topically instilled with NACA, were preserved and reduced, respectively, and are directly related to reduced *Txnip* and increased thioredoxin (*Trx*) expression levels. Overall, our findings demonstrated the protective ability of NACA to abate aberrant redox-active pathways, particularly the ROS/TRX/TXNIP axis, thereby preventing cataractogenesis and preserving eye lens integrity and ultimately impeding aging-related cataracts.

## 1. Introduction

Cataracts remain the leading cause of blindness worldwide, with aging-induced cataracts accounting for the majority of cases [1,2]. As the global population ages, the burden of cataracts is expected to increase, creating an urgent need for effective preventive and therapeutic strategies. Currently, surgery is the only definitive treatment for cataracts; however, its high cost, limited accessibility in resource-constrained settings, and associated surgical risks necessitate the development of alternative noninvasive approaches. Clinically, age-related cataracts are categorized into three primary types based on the morphological location of the cataract: nuclear, cortical, and posterior subcapsular. Nuclear cataracts involve progressive opacification of the central lens nucleus, typically driven by chronic oxidative stress and protein aggregation. Cortical cataracts originate in the lens cortex, whereas posterior subcapsular cataracts (PSCs) arise beneath the posterior lens capsule. Each type exhibits distinct pathological features and underlying mechanisms, with oxidative stress recognized as a critical contributing factor, particularly in cortical and nuclear cataract formation [3,4,5].

The decline in genetically encoded protective functions of antioxidant molecules, induced by aging or oxidative stress, contributes to age-related disorders, including vision-threatening diseases. Young, healthy cells possess well-regulated antioxidant systems that effectively protect them against various stressors. However, these protective mechanisms become compromised as age advances, leading to an increased susceptibility to oxidative stress-related pathological conditions [6,7,8]. Oxidative stress is widely recognized as a key factor in cataractogenesis [3,4,9,10,11]. Reactive oxygen species (ROS) generated in the lens due to aging, environmental factors, or metabolic disturbances can cause oxidative damage to proteins, lipids, and DNA. ROS are produced as natural byproducts through cellular metabolism, and their physiological levels are tightly controlled by antioxidant molecules. Although ROS are generally regarded as harmful molecules causing cellular damage, physiological levels of ROS play essential roles in maintaining survival-associated cell signaling, cellular homeostasis, and cell integrity [12,13,14]. However, excessive ROS production, particularly in response to defective metabolic machinery like mitochondrial activity or environmental stressors, can disrupt the cell’s antioxidant system leading to cellular damage and disorders, including cataracts [15,16]. Thus, the biological consequences of ROS largely depend on their intracellular concentrations; although moderate physiological levels of ROS are required for cell signaling and survival, excessive ROS accumulation leads to oxidative stress, resulting in cellular damage and apoptosis [17]. In this study, to evaluate the protective effects of the antioxidant N-acetylcysteine amide (NACA), concentrations were selected that were non-toxic yet sufficient to mitigate excessive ROS induced by hydrogen peroxide (H_2_O_2_) or tert-butyl hydroperoxide (tBHP). This oxidative insult compromises the structural and functional integrity of the lens, leading to protein aggregation, crystallin modification, and lens opacity. Although the human lens is well equipped with radical scavengers, including glutathione (GSH), superoxide dismutase, catalase, thioredoxin (TRX), peroxiredoxin 6 (PRDX6), and ascorbic acid and has high amounts of the chaperoning protein α-crystallin and efficient ultraviolet filters, oxidative stress will, in the long run, inevitably lead to lipid peroxidation and the formation and accumulation of crosslinked high-molecular-weight proteins and chromophores in the cortex and nucleus of the lens [4,5]. This accumulation results in enhanced light scatter and coloration. Furthermore, local rupture of membranes induces permeability changes, Ca^2+^ influx, disturbance of water homeostasis, and swelling, ultimately increasing light scattering and aggravating lens opacity. Aging diminishes the efficacy of these antioxidative systems, making the lens susceptible to oxidative damage.

In addition to oxidative stress, inflammation has emerged as a contributing factor to cataractogenesis. ROS overproduction can induce inflammation and vice versa. The nucleotide-binding oligomerization domain-like receptor family pyrin domain containing 3 (NLRP3) inflammasome has been implicated in oxidative stress and aging-related inflammation in lens epithelial cells (LECs) [17]. ROS can promote NLRP3 inflammasome activation in lenses [17,18], and its involvement in cataract formation has been previously demonstrated [19]. Thus, targeting the NLRP3 inflammasome reduced LEC damage triggered by H_2_O_2_ and lipopolysaccharide exposure in in vitro assays [18] and inhibited cataract formation in mice [19]. Recent studies revealed that antioxidants, such as N-acetylcysteine (NAC) and PRDX6, can impair the NLRP3 inflammasome activation pathway [17,20,21]. The activation of this pathway is driven by thioredoxin-interacting protein (TXNIP), a redox-sensitive mediator that amplifies oxidative damage through ROS production and inflammasome activation. TXNIP, the negative regulator of the antioxidant TRX, may dissociate in a time-dependent manner and bind to NLRP3, leading to inflammasome formation and activation. Since then, multiple studies have confirmed that TXNIP is required for NLRP3 inflammasome activation [22,23,24]. The dual role of TXNIP in oxidative stress and inflammation underscores its significance as a therapeutic target for cataract prevention [21].

Antioxidants have garnered attention as potential therapeutic agents for counteracting oxidative stress-induced cataracts. Among them, NAC and its derivative, NACA, have shown significant promise. NAC is a well-established antioxidant that replenishes intracellular GSH levels and directly scavenges ROS. However, its clinical application is limited by low bioavailability and suboptimal cell permeability. With its enhanced lipophilicity and cellular uptake, NACA overcomes these limitations and provides superior efficacy in reducing oxidative stress. Additionally, NACA has demonstrated the ability to modulate redox-sensitive pathways, including the suppression of TXNIP expression, suggesting broader protective mechanisms beyond direct ROS scavenging.

Preclinical studies have shown that NAC and NACA effectively prevent cataract formation in various induced cataract models. NAC delays cataract progression in selenite- and hyperoxia-induced cataract models by maintaining GSH levels and inhibiting oxidative damage markers. Similarly, NACA inhibits cataract formation in buthionine sulfoximine-induced cataract models by restoring GSH levels and reducing protein oxidation. However, the effects of NAC and NACA on natural aging-related cataracts, which mimic the gradual oxidative and inflammatory changes observed in human cataracts, remain underexplored.

This study aims to comprehensively evaluate the protective effects of NACA against oxidative stress-induced cataractogenesis using in vitro, ex vivo, and in vivo models. In particular, this study focuses on the modulation of TXNIP expression, ROS scavenging, and the preservation of lens transparency and structure. By elucidating the molecular mechanisms underlying NACA’s protective effects, this study seeks to provide critical insights into the development of antioxidant-based strategies for preventing aging-related cataracts and improving ocular health.

## 2. Materials and Methods

### 2.1. Chemicals

The following chemicals were used in this study: hydrogen peroxide (H_2_O_2_) (Fuji Film/WAKO, Osaka, Japan), NACA (Sigma-Aldrich, St. Louis, MO, USA), and NAC (Sigma-Aldrich). Medium 199 (Gibco, Thermo Fisher Scientific, Waltham, MA, USA) was used for ex vivo lens organ culture. For ROS measurement, the ROS Assay Kit dichlorodihydrofluorescein diacetate (H2-DCF-DA) was obtained from Invitrogen (Thermo Fisher Scientific), and tBHP solution was purchased from Sigma-Aldrich. All chemicals were prepared according to the manufacturer’s instructions, and stock solutions were diluted in sterile water or physiological saline (PS) as appropriate. The concentrations of the chemicals used in each experiment were optimized based on preliminary studies to ensure their efficacy and safety.

### 2.2. Cell Culture

Primary cultured mouse lens epithelial cells (MLECs) were isolated from 6-week-old BALB/c mice (N = 8) using previously described methods [25]. MLECs were maintained in Dulbecco’s modified Eagle’s medium (DMEM; Fuji Film/WAKO) supplemented with 10% fetal bovine serum (FBS; Sigma-Aldrich) at 37 °C in an air/CO_2_ (19:1) atmosphere, as previously described [26]. Cells from the third to fifth passages were utilized for the experiments. Simian virus 40-transformed human lens epithelial cells (HLECs; SRA01/04) were generously provided by Dr. Nobuhiro Ibaraki (formerly Ibaraki Eye Clinic, Tochigi, Japan). HLECs were cultured in DMEM supplemented with 20% FBS.

### 2.3. Measurement of ROS in MLECs and HLECs

MLECs and HLECs were cultured in 96-well plates (7500 and 5000 cells/well, respectively) in the presence or absence of 200 µM NACA for 1 h. Following pre-culture, cells were treated with H_2_O_2_ or tBHP solution at final concentrations of 0, 100, or 200 µM in the presence or absence of 200 µM NACA for 3 h to induce oxidative stress. The H_2_O_2_ and tBHP concentrations used in the present study were chosen based on well-established protocols that reliably induce oxidative stress in LECs. Specifically, H_2_O_2_ concentrations ranging from 50 to 200 µM have been validated to consistently elevate intracellular ROS levels in LECs, effectively mimicking oxidative stress conditions relevant to cataractogenesis [27,28]. Similarly, concentrations of tBHP between 30 and 300 µM have been previously demonstrated to robustly generate ROS and trigger relevant oxidative responses in LECs [29]. Thus, these conditions represent established and physiologically relevant oxidative stress models appropriate for studies of cataract pathophysiology.

Intracellular ROS levels were quantified using a fluorescent dye, H2-DCF-DA, according to our published protocol [30]. On the day of the experiment, the medium was replaced with Hank’s solution containing 10 μM of H2-DCF-DA dye. After 30 min, intracellular fluorescence was detected by excitation at 485 nm/emission at 530 nm using a Spectra Max Gemini EM (Molecular Devices, Sunnyvale, CA, USA). The experiment was performed in triplicate for each condition, and the data were normalized to untreated control cells.

### 2.4. Cell Survival Assay

Cell survival was assessed using the MTS assay (CellTiter 96^®^ AQueous One Solution Cell Proliferation Assay, Promega, Madison, WI, USA), according to the manufacturer’s instructions. MLECs and HLECs were used in these experiments. Briefly, the cells were seeded in a 96-well plate at a density of 5 × 10^3^ cells/well and cultured overnight to allow for attachment. MLECs or HLECs were then treated with either 0, 50, or 100 µM H_2_O_2_ in the presence or absence of 200 µM NACA for 4 h. The absorbance of each well was measured at 490 nm wavelength using a microplate reader. Cell survival was calculated as the percentage of the absorbance of treated cells relative to that of untreated control cells, which was set to 100%.

### 2.5. Real-Time Reverse Transcriptase-Quantitative Polymerase Chain Reaction (RT-qPCR)

To investigate the effects of NACA on H_2_O_2_-induced oxidative stress, MLECs were plated in triplicate into 35 mm culture dishes. Cells were cultured in DMEM containing 1% FBS in the presence of 0–100 μM NACA. Total RNA was extracted from MLECs using a miRNeasy Micro Kit (Qiagen, Valencia, CA, USA), according to the manufacturer’s protocol. To quantify the mRNA expression of the *Prdx6* and catalase genes, relative quantification of mRNA was performed using an Applied Biosystems^®^ 7300 system (Thermo Fisher Scientific). PCR amplification was conducted using TaqMan Universal Master Mix and a predeveloped mouse *Prdx6* (Assay ID: Mm00725435), mouse catalase (Assay ID: Mm:00437992), mouse *Txnip* (Assay ID: Mm00726847), and mouse *Nlrp3* (Assay ID: Mm04210224) probes (Thermo Fisher Scientific). The relative quantities of *Prdx6* and catalase mRNA were determined using the comparative Ct method and subsequently normalized using a predeveloped eukaryotic 18S rRNA endogenous control (Assay ID: Hs99999901; Thermo Fisher Scientific).

### 2.6. Animals

All procedures involving animals (in vivo and ex vivo) were conducted in accordance with institutional guidelines and were approved by the Committee of Animal Research at Kanazawa Medical University (approval number [2024-33]). In this study, 12-week-old female Sprague–Dawley rats (for ex vivo experiments) and 62-week-old C57BL/6 female mice (for in vivo experiments) were used. Animals were randomly assigned to experimental groups to minimize bias. Animals displaying pre-existing lens opacity or other ocular abnormalities before the start of the experiment were excluded from the study. Additionally, any animals that exhibited signs of systemic illness or significant weight loss exceeding 10% of their body weight during the study period were excluded to prevent confounding effects. For the ex vivo experiments, lenses that were damaged during the dissection process were also excluded from analysis. All animals were housed under standard laboratory conditions with a 12-hour light/dark cycle and free access to food and water. Before the start of experiments, animals were acclimatized to the experimental facility for at least 7 days to minimize the effects of transport-induced stress.

Regarding data analysis, all data points were included unless clear technical errors were identified. Exclusions were only made in cases where lens integrity was not maintained during ex vivo culture, optical imaging artifacts prevented accurate assessment of lens opacity, or equipment malfunctions occurred during fluorescence or absorbance measurements. No animals were removed from the in vivo study after initial inclusion.

### 2.7. Ex Vivo Lens Organ Culture and Imaging

**Figure 1:**
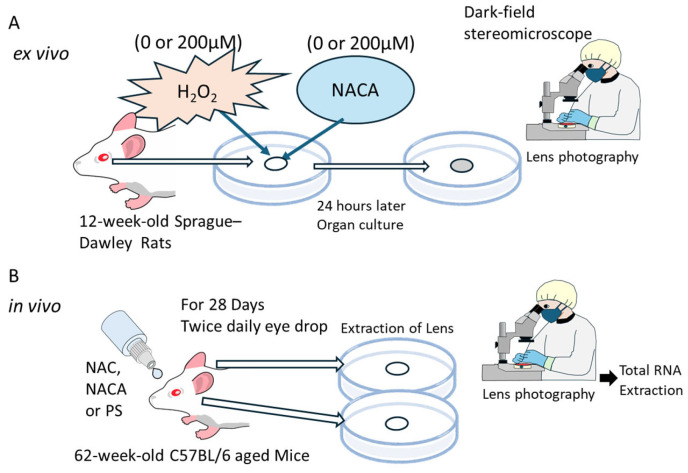
Experimental design for the ex vivo lens organ culture and in vivo cataract suppression studies. (**A**) Ex vivo organ culture: To evaluate oxidative stress and the effects of NACA on lens opacity, lenses were cultured for 24 h with either 0 or 100 µM H_2_O_2_ in the presence or absence of 200 µM NACA. Lens opacity was assessed using a stereoscopic microscope with dark-field illumination, and opacity levels were quantified. (**B**) In vivo cataract suppression study: Groups of 62-week-old C57BL/6 mice (N = 6 per group) received eye drops containing PS (0.9%, pH 7.0; control), 2 mM NAC, or 2 mM NACA twice daily for 4 weeks. Lenses were extracted at the end of the treatment period, and the opacity was evaluated using a dark-field stereoscopic microscope. Lens capsules were used for total RNA extraction to analyze the expression of oxidative stress-related genes, including *Prdx6*, *Txn1*, and *Txnip*, by RT-qPCR. H_2_O_2_, hydrogen peroxide; NACA, N-acetylcysteine; PRDX6, peroxiredoxin 6; PS, physiological saline; RT-qPCR, real-time reverse transcriptase-quantitative polymerase chain reaction; TXN1, thioredoxin-1; TXNIP, thioredoxin-interacting protein expression.

The lenses were excised from 12-week-old rats (N = 26 eyes) and subjected to ex vivo organ culture. The lenses were maintained in Medium 199 at 37 °C in a humidified atmosphere with 5% CO₂. To evaluate the effects of oxidative stress and antioxidant treatment, the culture medium was supplemented with 0 or 100 µM H_2_O_2_ in the presence or absence of 200 µM NACA, for 24 h. Following the treatment, the lenses were photographed using a stereoscopic microscope with dark-field illumination (SMZ745T, Nikon Instech, Tokyo, Japan), as illustrated in Figure 1A. Lens opacity was quantified by analyzing digital images of the lenses using the Image Quant™ TL analysis software (Cytiva, Global Life Sciences Technologies Japan K.K., Tokyo, Japan).

### 2.8. In Vivo Experiments on the Suppression of Aging-Related Cataracts by Topical Administration of NAC and NACA

Aged C57BL/6 mice (62 weeks old; N = 6 per group) were purchased from Jackson Laboratory Japan, Inc. (Yokohama, Japan). Eye-drop solutions containing 0.9% PS (pH 7.0, control), 2 mM NAC, or 2 mM NACA were prepared. A concentration of 2 mM was determined based on preliminary experiments using a small number of mice, which demonstrated optimal effects on lens transparency without apparent toxicity (Figure S1). Each solution (20 µL per dose) was administered to mice twice daily for 28 consecutive days, as illustrated in Figure 1B. At the end of the treatment period, the mice were sacrificed by CO₂ inhalation, and their lenses were carefully excised. Subsequently, the lenses were photographed using a stereoscopic microscope with dark-field illumination, as described in Section 2.6.

For molecular analyses, lens capsules containing epithelial cells were dissected, and total RNA was extracted using a miRNeasy Micro Kit (Qiagen). PCR amplification was conducted using TaqMan Universal Master Mix and a predeveloped mouse *Prdx6* (Assay ID: Mm00725435), thioredoxin-1 (*Txn1*) (Assay ID: Mm00726847), and *Txnip* (Assay ID: Mm00726847) probe mix (Thermo Fisher Scientific). The relative quantities of each gene were determined using the comparative Ct method and subsequently normalized using a predeveloped TaqMan mouse glyceraldehyde 3-phosphate dehydrogenase (*Gapdh*) control reagent VIC probe (Assay ID: Mm99999915) as an endogenous control (Thermo Fisher Scientific).

### 2.9. Statistical Analysis

All statistical analyses were performed using SigmaPlot 15.0 software (Systat Software, San Jose, CA, USA). One-way analysis of variance (ANOVA) followed by Tukey’s post hoc test was used to compare multiple groups. Data are presented as the mean ± standard deviation (SD), and statistical significance was set at *p* < 0.05.

### 2.10. Application of Artificial Intelligence in Writing Assistance

In this study, we used Paperpal (2.15.13, developed by Cactus Communications, Mumbai, India), an AI-powered writing assistant, to improve the grammar, language, and clarity of the manuscript. No content was generated by artificial intelligence, and all intellectual contributions, including data analysis, interpretation, and conclusions, were made solely by the authors.

## 3. Results

### 3.1. Effects of NACA on Cellular Viability in MLECs and HLECs Exposed to H_2_O_2_-Mediated Oxidative Stress

**Figure 2:**
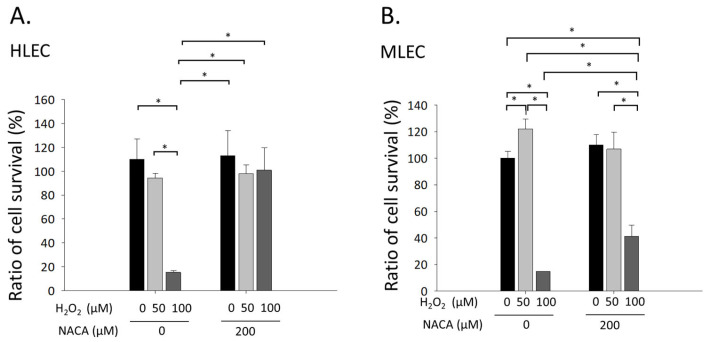
Effects of NACA on the viability of HLECs and MLECs under H_2_O_2_-induced oxidative stress. (**A**) HLECs and (**B**) MLECs exposed to 50 or 100 µM H_2_O_2_ in the presence or absence of 200 µM NACA. At the concentration of 50 µM H_2_O_2_, both HLECs and MLECs did not show increased cell death. Exposure to 100 µM H_2_O_2_ significantly reduced the viability of both LECs compared with that of untreated controls (* *p* < 0.001). However, co-treatment with 200 µM NACA significantly mitigated the H_2_O_2_-induced reduction in cell viability compared with that in cells treated with H_2_O_2_ alone (* *p* < 0.01. Data are presented as the mean ± SD of three independent experiments, each performed in triplicate. Statistical analysis was performed using one-way ANOVA with Tukey’s test. ANOVA, analysis of variance; HLEC, human lens epithelial cell; LEC, lens epithelial cell; MLEC, mouse lens epithelial cell; SD, standard deviation.

The influence of NACA on cellular viability during oxidative stress was investigated using the MTS assay in both MLECs and HLECs. Exposure to 100 µM H_2_O_2_ caused a statistically significant reduction in cellular viability compared with untreated controls in both cell types (* *p* < 0.001, ANOVA; Figure 2A,B). Notably, supplementation with 200 µM NACA significantly mitigated the H_2_O_2_-induced decline in cell viability when juxtaposed with cells treated solely with H_2_O_2_ (* *p* < 0.001; Figure 2A,B). These observations demonstrate that NACA exhibits substantial cytoprotective properties against H_2_O_2_-induced oxidative stress in MLECs and HLECs, lending credence to its potential application as an antioxidant in LECs.

### 3.2. Effects of NACA on ROS Levels in HLECs and MLECs Under H_2_O_2_- or tBHP-Induced Oxidative Stress

**Figure 3:**
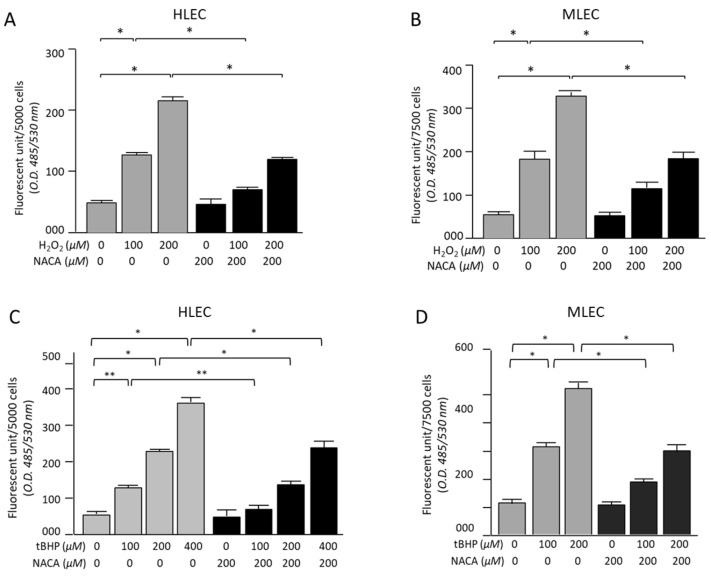
Effects of NACA on ROS levels in MLECs and HLECs under H_2_O_2_- and tBHP-induced oxidative stress. HLECs and MLECs were subjected to oxidative stress through exposure to H_2_O_2_ at 100 and 200 µM or tBHP at 0, 100, 200, and 400 µM. Experiments were conducted with and without 200 µM NACA (**A**–**D**). Both H_2_O_2_ and tBHP significantly decreased the viability of HLECs and MLECs compared to that of untreated control cells (* *p* < 0.001; ** *p* < 0.05, (**A**–**D**)). However, the addition of 200 µM NACA significantly mitigated the reduction in cell viability induced by H_2_O_2_ and tBHP, compared to cells treated with these oxidants alone (* *p* < 0.001; ** *p* < 0.05, (**A**–**D**)). In MLECs, 0–200 µM concentration of tBHP was selected because 400 µM tBHP was cytotoxic (**D**). The results are expressed as the mean ± SD of three independent experiments, each performed in triplicate. Statistical analysis was conducted using a one-way ANOVA with Tukey’s post hoc test. ROS, reactive oxygen species; tBHP, tert-butyl hydroperoxide.

Intracellular ROS levels were measured in HLECs and MLECs after exposure to H_2_O_2_- or tBHP-induced oxidative stress (Figure 3A–D). The results demonstrated a concentration-dependent increase in ROS production in the treatment groups. Specifically, both MLECs and HLECs treated with H_2_O_2_ at final concentrations of 100 µM and 200 µM exhibited a significant increase in fluorescence intensity compared with untreated control cells, indicating elevated intracellular ROS levels (Figure 3A,B). A similar trend was observed in tBHP-treated cells, in which ROS levels increased proportionally with increasing concentrations of tBHP (Figure 3C,D). Importantly, the addition of 200 µM NACA significantly attenuated ROS production in both H_2_O_2_- and tBHP-treated cells (Figure 3). In the presence of NACA, intracellular ROS levels were markedly reduced compared with the respective levels without NACA treatment. The observed reduction in ROS levels was statistically significant (* *p* < 0.001 for both H_2_O_2_ and tBHP at 200 µM, ** *p* < 0.05 for tBHP at 100 µM, ANOVA; Figure 3). These findings indicate that NACA effectively mitigated oxidative stress-induced ROS accumulation in both MLECs and HLECs.

### 3.3. Effects of NACA on Prdx6 and Catalase mRNA Expression Under H_2_O_2_-Induced Oxidative Stress

**Figure 4:**
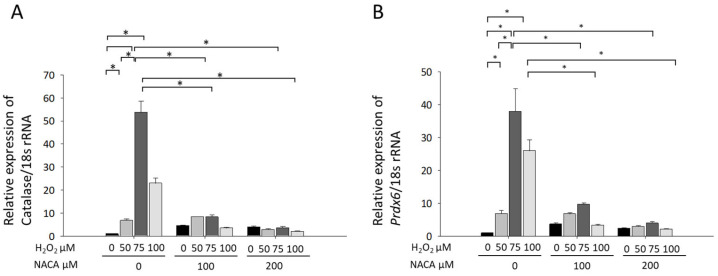
Effects of NACA on *Prdx6* and catalase mRNA expression under H_2_O_2_-induced oxidative stress. RT-qPCR was employed to measure the mRNA expression levels of (**A**) *Prdx6* and (**B**) catalase in MLECs treated with 50–100 µM H_2_O_2_, with or without 100 or 200 µM NACA. The results showed a significant increase in the mRNA expression of both *Prdx6* and catalase in cells exposed to H_2_O_2_, compared with that in untreated control cells (* *p* < 0.001). Notably, the addition of NACA at either 100 µM or 200 µM significantly reduced the H_2_O_2_-induced elevation of these antioxidant enzymes (* *p* < 0.001). The findings are presented as the mean ± SD of three separate experiments, each conducted in triplicate. One-way ANOVA followed by Tukey’s post hoc test was used for statistical analysis.

To investigate the effects of NACA on oxidative stress-induced gene expression, *Prdx6* and catalase mRNA levels were quantified using RT-qPCR. Treatment with 50–100 µM H_2_O_2_ significantly increased the mRNA expression of both *Prdx6* and catalase compared with that in untreated controls (Figure 4A,B), corroborating our study findings on the induction of these endogenous antioxidant enzymes under oxidative stress conditions [30,31]. However, co-treatment with 100 and 200 µM NACA significantly suppressed the H_2_O_2_-induced upregulation of *Prdx6* and catalase mRNA expression (* *p* < 0.001; ANOVA). These findings suggest that NACA mitigates oxidative stress through its ROS-scavenging activity, thereby inhibiting *Prdx6* and catalase upregulation. Both enzymes play critical roles in the antioxidant defense system of the lens and are typically induced in response to oxidative stress. The observed suppression of their mRNA expression by NACA may be attributed to its capacity to reduce intracellular ROS levels, thereby counteracting H_2_O_2_-induced oxidative stress.

### 3.4. Effects of NACA on Lens Opacity Induced by H_2_O_2_ in Ex Vivo Organ Cultures

**Figure 5:**
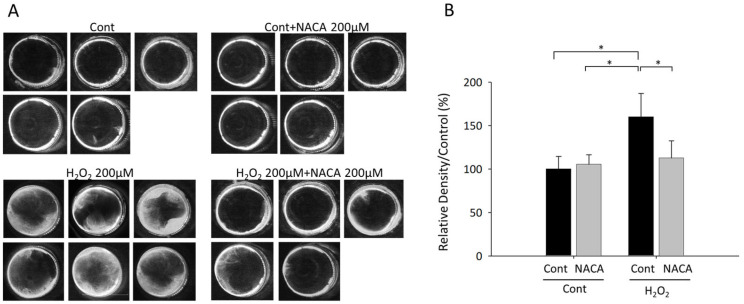
Effects of NACA on lens opacity induced by H_2_O_2_ in ex vivo organ culture. Images of lenses from 12-week-old rats cultured ex vivo under various conditions were captured (**A**). A quantitative evaluation of lens opacity under different treatment conditions was performed (**B**). Lenses cultivated without H_2_O_2_ exposure, irrespective of the presence or absence of 200 µM NACA, exhibited no visible opacity (N = 5 for each group, (**A**,**B**)). However, lenses treated with 100 µM H_2_O_2_ demonstrated considerable opacity, establishing a reliable model for oxidative stress-induced cataracts (N = 5, * *p* < 0.001, (**A**,**B**)). The addition of 200 µM NACA significantly decreased H_2_O_2_-induced lens opacity (N = 6, * *p* < 0.001, **A**,**B**). A one-way ANOVA revealed a statistically significant difference among the treatment groups (F (3,17) = 11.466, *p* < 0.001), indicating that NACA treatment significantly influenced oxidative stress levels in LECs. The effect size, calculated as η^2^ = 0.669, suggests that 66.9% of the total variance can be attributed to the treatment conditions, indicating a large effect. Post hoc Tukey’s test showed significant differences between the H_2_O_2_-treated and control groups (*p* < 0.001), as well as between the H_2_O_2_-treated and H_2_O_2_ + NACA-treated groups (*p* = 0.004). However, no significant difference was found between the control and NACA-treated control groups (*p* = 0.971). Confidence intervals (95% CI) for group comparisons were not explicitly calculated in this study, but future research should include CI estimates to better interpret the precision of the observed differences. These findings demonstrate the efficacy of NACA in mitigating oxidative stress-induced lens opacity, suggesting its potential as a therapeutic agent to prevent cataract formation. CI, confidence interval.

The impact of oxidative stress and antioxidant intervention on lens transparency was investigated using ex vivo cultured lenses from 12-week-old Sprague–Dawley rats under diverse conditions. Exposure to 100 µM H_2_O_2_ resulted in a statistically significant increase in lens opacity compared with that of untreated controls (* *p* < 0.001; ANOVA), thereby establishing a valid oxidative stress-induced cataract model. Notably, concurrent administration of 200 µM NACA substantially reduced H_2_O_2_-induced lens opacity (* *p* < 0.001; ANOVA), as illustrated in Figure 5A,B. These observations suggest that NACA effectively counteracts oxidative stress-induced lens opacity, presumably through its ROS scavenging capabilities. These findings underscore the therapeutic potential of NACA in preventing oxidative stress-induced cataract formation.

### 3.5. Effects of NAC and NACA Eye Drops on Age-Related Cataract and Oxidative Stress in Aged Mice In Vivo

**Figure 6:**
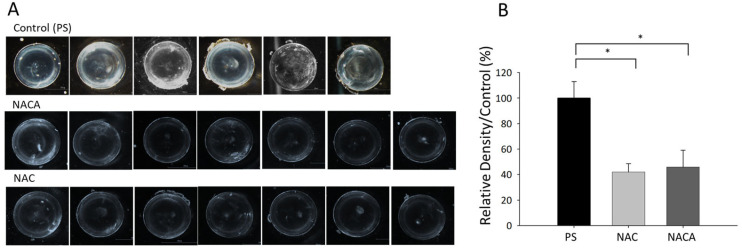
Effects of NAC and NACA on lens opacity in aged mice. (**A**) Representative images of lenses from 62-week-old C57BL/6 mice treated with PS (control, N = 6; 6 eyes), 2 mM NAC (N = 7; 7 eyes), or 2 mM NACA (N = 7; 7 eyes) for 4 weeks. Lens opacity was assessed under a dark-field stereoscopic microscope. Lenses from the PS-treated group exhibited significant opacity, whereas lenses from the NAC- and NACA-treated groups demonstrated markedly reduced opacity. (**B**) Quantitative analysis of lens opacity in PS-, NAC-, and NACA-treated groups. NAC and NACA treatment significantly reduced lens opacity compared with the PS-treated group (*p < 0.001). Data are presented as the mean ± SD. A one-way ANOVA revealed a statistically significant difference among the treatment groups (F (2,15) = 40.482, *p < 0.001), indicating that NAC and NACA treatments significantly influenced the measured parameter. The effect size, calculated as η^2^ = 0.844, suggests that 84.4% of the total variance can be attributed to the treatment conditions, demonstrating a very large effect. Post hoc Tukey’s test confirmed significant differences between the PS-treated group and both the NAC-treated group (*p < 0.001) and the NACA-treated group (*p* < 0.001), suggesting that both antioxidant treatments significantly reduced the measured parameter compared to the control. However, no statistically significant difference was observed between the NAC and NACA groups (*p* = 0.808), indicating that their effects were equivalent in this experimental setting. Confidence intervals (95% CI) for group comparisons were not explicitly calculated in this study. However, future research should include CI estimates to improve the precision of the reported differences and provide a clearer interpretation of the variability in treatment effects. NAC, N-acetylcysteine.

Following a 28-day treatment period with PS, lenses of 62-week-old mice in the control group exhibited significant opacity, as observed under dark-field illumination (* *p* < 0.001, ANOVA; Figure 6A). In contrast, mice treated with 2 mM NAC or 2 mM NACA demonstrated marked suppression of lens opacity. The degrees of lens opacity in the NAC- and NACA-treated groups were significantly lower than that in the PS-treated group (* *p* < 0.001; Figure 6B).

**Figure 7:**
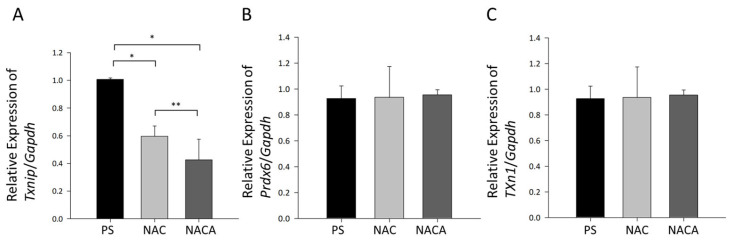
Effects of NAC and NACA on *Txnip* and antioxidant gene expression in aged mice. *Txnip* mRNA expression in LECs of 62-week-old C57BL/6 mice treated with PS (control), 2 mM NAC, or 2 mM NACA for 4 weeks. NAC and NACA significantly downregulated *Txnip* mRNA expression levels compared with those in the PS-treated group (* *p* < 0.001, ** *p* < 0.05) (**A**). The expression levels of endogenous antioxidants, *Prdx6* (**B**) and *Txn1* (**C**), in LECs. No significant differences were observed among the PS-, NAC-, and NACA-treated groups (**B**,**C**). Data are presented as the mean ± SD from six mice per group (N = 6). Statistical analysis was conducted using one-way ANOVA followed by Tukey’s post hoc test. GAPDH, glyceraldehyde 3-phosphate dehydrogenase.

At the molecular level, *Txnip* mRNA expression, a regulatory factor involved in the oxidative stress response, was significantly downregulated in the NAC- and NACA-treated groups compared with that in the PS-treated group (* *p* < 0.01; ** *p* < 0.05, Figure 7A). This observation suggests that the antioxidant effects of NAC and NACA contribute to the suppression of oxidative stress and subsequent lens opacity. However, the expression of endogenous antioxidants, such as *Prdx6* and *Txn1*, in LECs remained unaltered following NAC or NACA treatment (Figure 7B,C).

These findings indicate that topical administration of NAC and NACA effectively suppressed aging-related cataract formation in aged mice by mitigating oxidative stress, as evidenced by the downregulation of *Txnip* mRNA. This protective effect is independent of changes in the expression of endogenous antioxidant enzymes such as PRDX6 and catalase.

## 4. Discussion

This study comprehensively examined the protective effects of NACA against oxidative stress-induced damage in LECs, ex vivo organ-cultured lenses, and an in vivo aging-related cataract model. These findings demonstrate that NACA exhibits significant efficacy in attenuating oxidative stress through its potent ROS-scavenging activity, suppression of oxidative stress-related gene expression, and preservation of lens transparency. These results provide valuable insights into the potential therapeutic applications of NACA in the prevention and management of cataracts.

Oxidative stress-induced cell death in LECs is a critical initiating event in cataractogenesis because these cells are essential for maintaining lens homeostasis and transparency [4,11]. The results of the MTS assay showed that exposure to 100 µM H_2_O_2_ significantly reduced the viability of both MLECs and HLECs, reflecting the detrimental effects of oxidative stress. However, the addition of 200 µM NACA significantly attenuated the reduced cell viability, indicating that NACA has a strong cytoprotective effect. This protective effect is likely attributed to NACA’s ROS-scavenging properties, which directly neutralize ROS generated under oxidative stress [32]. At the intermediate concentration of 50 µM H_2_O_2_, LECs did not show increased cell death; rather, they exhibited enhanced cell viability indicative of a hormetic response. This hormetic phenomenon, wherein low-level oxidative stress triggers adaptive protective mechanisms and promotes cellular survival, aligns with previously reported observations in LECs [30]. These findings underline the importance of considering concentration-dependent effects when interpreting oxidative stress outcomes. Our results revealed that NACA effectively preserved LEC viability under H_2_O_2_-induced oxidative stress and mitigated lens opacity in both ex vivo and in vivo models. These findings align with those of previous studies demonstrating the antioxidative and cytoprotective efficacy of NACA and related compounds in various ocular systems [32,33,34]. The enhanced efficacy of NACA compared to that of NAC can be attributed to its greater cell permeability and ability to replenish intracellular GSH levels in LECs [32,33]. Previous studies have clearly established that NACA readily permeates cell membranes and the blood–brain barrier, demonstrating effective cellular internalization [35]. By preserving cellular viability, NACA prevents the downstream consequences of oxidative stress, such as apoptosis, metabolic dysfunction, and, eventually, lens opacity. These results highlight the importance of antioxidant supplementation as a therapeutic strategy in cataract prevention.

Endogenous antioxidant enzymes, such as PRDX6 and catalase, play pivotal roles in mitigating oxidative damage in the lens [36,37,38]. The upregulation of these enzymes observed following H_2_O_2_ exposure aligns with their established function as a defense mechanism against oxidative stress [39,40]. However, co-treatment with NACA significantly suppressed the H_2_O_2_-induced increase in *Prdx6* and catalase mRNA expression in MLECs. Interestingly, at high concentrations of H_2_O_2_ (e.g., 100 μM), we observed a decrease in antioxidant gene expression in the absence of NACA. This phenomenon might seem counterintuitive, given that moderate oxidative stress typically activates Nrf2 signaling and promotes the expression of antioxidant genes. However, excessive oxidative stress can overwhelm cellular antioxidant defenses, potentially impairing Nrf2-dependent transcriptional activation by disrupting its nuclear localization or reducing its binding affinity to antioxidant response elements in gene promoters. In contrast, NACA treatment effectively reduces intracellular ROS to manageable levels, thereby preserving Nrf2 signaling capacity and stabilizing the expression of antioxidant genes, such as *Prdx6* and catalase. This finding suggests that NACA reduces intracellular ROS levels to a degree where upregulation of these antioxidant enzymes is no longer required. This finding is consistent with that of Martis et al., who demonstrated that NACA significantly increased GSH levels in the lens epithelium and cortex, mitigating oxidative stress and reducing the need for the compensatory upregulation of antioxidant enzymes [33]. Furthermore, Wang et al. observed that NAC restored catalase activity in hyperoxia-exposed rabbit lenses, indicating its role in preserving antioxidant defenses [41]. Interestingly, in our in vivo model, the expression levels of *Prdx6* and catalase remained unchanged after treatment with NAC or NACA. This discrepancy may be explained by the greater complexity of in vivo systems, in which multiple compensatory mechanisms may interact to maintain redox homeostasis. It also underscores the distinct regulatory roles of endogenous antioxidant enzymes and exogenous ROS scavengers such as NACA in oxidative stress management.

In our ex vivo organ culture model, treatment with 100 µM H_2_O_2_ induced significant lens opacity, demonstrating its reliability as an oxidative stress-induced cataract model. NACA treatment significantly attenuated this opacity, suggesting that it effectively counteracted oxidative stress in the lens, which was consistent with the findings of Martis et al. that NACA (10 mM) reduced lens opacity in porcine lenses exposed to H_2_O_2_ and glucose oxidase [33]. Additionally, NACA-treated lenses have increased cysteine and GSH levels, which likely contributed to the observed reduction in oxidative damage and opacity [33]. These results aligned with those of the in vivo experiments using 62-week-old mice. Lenses from the control group treated with PS displayed marked opacity along with structural abnormalities in the superficial cortical fibers. Conversely, mice treated with NAC or NACA exhibited significantly reduced lens opacity. A previous study revealed that in aging lenses or lenses under oxidative stress, the size of the GSH pool is diminished, and some protein thiols are S-thiolated by oxidized nonprotein thiols to form protein-thiol mixed disulfides, either as protein-S-S-glutathione or protein-S-S-cysteine [3,42]. The mechanism underlying aging-related cataracts has been hypothesized to be the oxidation-induced protein-thiol with disulfide formation, such as protein-S-S-glutathione and protein-S-S-cysteine mixed disulfides, which, if not reduced promptly, can change the protein conformation to allow cascading modifications of various types, leading to protein–protein aggregation and insolubilization [3,42]. Furthermore, NAC is an efficient reducing agent for protein disulfide through the classic thiol–disulfide interchange mechanism [43,44]. Thus, our findings suggest that NAC and NACA not only reduce oxidative stress but also preserve the structural integrity of the lens, likely by preventing ROS-induced protein damage and aggregation of lens crystalline. The H_2_O_2_-induced cataract model is a valuable tool for studying the oxidative stress-related mechanisms in cataractogenesis [45]. It closely mimics the key aspects of aging-related cataracts, including GSH depletion, ROS accumulation, and protein aggregation [45]. However, its utility is best paired with natural aging models such as C57BL/6 aged mice to provide a comprehensive understanding of cataract formation and potential therapeutic strategies.

Previous in vivo studies have demonstrated the efficacy of NAC and NACA in preventing oxidative stress-induced cataract formation. For example, in an L-buthionine-(S,R)-sulfoximine (BSO; a GSH synthesis inhibitor)-induced cataract model, NACA significantly inhibited cataract formation, with 80% of NACA-treated Wistar rats showing clear lenses compared with the BSO-only group, in which all lenses developed severe opacities [46]. This protection is attributed to the ability of NACA to restore GSH levels, reduce protein carbonylation, and limit lipid peroxidation [46]. Similarly, NAC delayed cataract progression in rat models of selenite-induced cataracts by maintaining GSH levels and reducing oxidative damage markers, such as MDA and protein oxidation [47]. Our study introduces a critical innovation by utilizing a mouse model of age-related cataracts rather than relying on models with induced oxidative stress. This age-related model more accurately mimics the natural progression of age-related cataracts in humans, which is characterized by a gradual decline in antioxidant defenses, GSH depletion, and cumulative protein aggregation [48,49,50]. Unlike acute oxidative stress-induced models (including BSO- or selenite-induced cataracts), age-related cataract models allow for the investigation of the long-term effects of antioxidants, such as NAC and NACA, under physiologically relevant conditions.

In this context, our findings demonstrated that both NAC and NACA effectively reduced lens opacity in aged mice. However, a notable difference was observed in their molecular effects; although both NAC and NACA suppressed the expression of *Txnip* mRNA, a key mediator of oxidative stress and apoptosis, NACA exhibited a significantly stronger effect. This suggests that the superior ability of NACA to modulate redox-sensitive signaling pathways, in addition to its potent ROS-scavenging activity, may provide unique advantages in managing age-related cataracts.

The most compelling finding of this study was the significant downregulation of *Txnip* mRNA expression in lenses treated with NAC and NACA compared with that in controls. TXNIP, a critical regulator of oxidative stress and inflammation, promotes ROS production and activates the NLRP3 inflammasome [51,52]. A previous study demonstrated that NAC effectively reduces TXNIP and NLRP3 expression in a microglial inflammation model, highlighting its anti-inflammatory properties [21]. Several studies have shown that antioxidants like acetaminophen or NAC, including dietary supplements, can blunt the lipopolysaccharide- or oxidative-stimulated NLRP3 inflammasome activation in vitro and in vivo [20,21,53,54,55]. In the current study, the stronger suppression of *Txnip* expression in LECs by NACA offers a mechanistic explanation for its ability to mitigate oxidative stress and inhibit cataract formation in vivo. Tosi et al. discussed oxidative damage in retinal pigment epithelial cells caused by ROS, which contributes to conditions such as age-related macular degeneration [56]. TXNIP is implicated in these processes because its overexpression can impair antioxidant defenses in the retina. N-acetyl-L-cysteine ethyl ester (NACET) demonstrated superior efficacy in reducing TXNIP expression and oxidative stress compared to NAC, thereby protecting retinal pigment epithelium cells and enhancing GSH levels [56]. Among these, NACET and NACA appear to be more effective due to their superior pharmacokinetic properties, making them potential candidates for treating a wide range of oxidative stress-induced ocular diseases.

Another compelling finding from this study is the significant downregulation of *Txnip* mRNA expression in lenses treated with NAC and NACA compared with that in untreated controls. TXNIP is well-established as a key regulator of oxidative stress and inflammation, known to promote ROS production and activate the NLRP3 inflammasome [21,50,51]. Although some reports have indicated that high-glucose conditions can upregulate *Txnip* expression in LECs [57,58], no direct evidence currently links *Txnip* expression specifically to age-related cataractogenesis. Nevertheless, evidence from other biological systems clearly indicates that *Txnip* expression significantly increases under conditions of aging, oxidative stress, inflammation, or pathologies [59,60,61]. For example, Lin et al. demonstrated that TXNIP exacerbates senescence via activation of the CDK-Rb pathway under oxidative stress conditions [59], highlighting its potential relevance to age-related pathologies.

Our group is actively investigating the role of the ROS/TXNIP/TRX axis specifically within the context of lens biology, and preliminary data indicate promising correlations between this pathway and antioxidant regulatory mechanisms in the lens. Although our findings strongly suggest that suppression of *Txnip* expression may significantly contribute to lens protection, further mechanistic studies are required to establish a definitive link with age-related cataract formation.

Supporting our observations, NAC effectively reduces TXNIP and NLRP3 inflammasome expression in models of microglial inflammation [22]. Similarly, various antioxidants—including acetaminophen, NAC, and dietary supplements—have demonstrated efficacy in inhibiting inflammasome activation induced by oxidative and inflammatory stimuli both in vitro and in vivo [21,22,52,53,54]. Recent studies, such as Tosi et al. [56], also highlight oxidative damage mediated by TXNIP in retinal pathologies, including age-related macular degeneration. These retinal models indicate that NACET possesses superior pharmacokinetic properties compared to NAC, significantly reducing *Txnip* expression, alleviating oxidative stress, and protecting retinal pigment epithelial cells by enhancing intracellular GSH levels [56]. Thus, pharmacokinetically optimized antioxidants such as NACET and NACA may represent promising therapeutic candidates for the prevention and treatment of oxidative stress-driven ocular diseases, including cataracts.

While prior studies have highlighted the superior antioxidant efficacy of NACA to NAC in ex vivo and in vitro cataract models [33,41], our in vivo results revealed no significant differences between NAC and NACA in suppressing lens opacity in aged mouse lenses. However, a notable difference was observed in the suppression of *Txnip* expression, with NACA demonstrating a greater ability to downregulate *Txnip* levels than NAC. This suggests that the two antioxidants may exhibit divergent mechanisms of action at the molecular level, particularly in modulating the oxidative stress signaling pathways. The unaltered expression of *Prdx6* and catalase in the in vivo model, despite the suppression of *Txnip*, further emphasizes the independence of NACA’s protective effects from endogenous antioxidant enzyme regulation. This finding suggests that NACA primarily exerts its effects by reducing the initial oxidative burden, thereby preventing activation of stress-responsive pathways. While this study demonstrates that NACA downregulates *Txnip* expression, the precise molecular mechanisms underlying this suppression remain unclear. Whether NACA directly influences *Txnip* transcription or indirectly modulates it through other redox-sensitive signaling pathways warrants further investigation. Additionally, the downstream effects of *Txnip* suppression, including its effects on inflammasome activation and apoptotic pathways, should be explored in greater detail.

Regarding the complexity of cataract phenotypes, it is important to acknowledge that age-related cataracts primarily consist of nuclear, cortical, and posterior subcapsular types, each exhibiting distinct morphological characteristics and pathophysiological mechanisms. Nuclear cataracts, in particular, predominantly result from chronic oxidative damage and subsequent protein aggregation occurring in the lens nucleus. Oxidative stress is widely accepted as a major contributing factor in nuclear cataract formation, supported by evidence linking prolonged oxygen exposure, such as hyperbaric oxygen therapy and vitrectomy, to increased incidence of nuclear cataracts [62,63,64]. Elevated oxygen levels following vitrectomy, for instance, persistently increase oxidative stress within the lens nucleus, exacerbating protein aggregation and lens opacity [3,64]. Therefore, a critical question remains whether antioxidants such as NACA, which showed efficacy against oxidative stress-induced and age-related cortical cataracts in our study, can effectively penetrate and protect the lens nucleus. Although our findings demonstrate robust antioxidant and signaling modulation capacities of NACA, additional studies investigating pharmacokinetic profiles and antioxidant penetration into deeper lens regions are essential to conclusively determine its potential therapeutic efficacy against nuclear cataracts. A limitation of our current study is that the quantitative assessments of the lens were conducted by measuring whole-lens opacity without differentiating specific lens regions such as the nucleus, cortex, or posterior subcapsular areas. Although visual examinations of lens images (Figure 6A) suggest a reduced nuclear opacity in NAC- and NACA-treated groups compared to that in the control group, our methodology did not permit region-specific quantitative analyses. Therefore, future studies utilizing advanced imaging techniques to separately quantify opacity within different lens regions are necessary to conclusively determine whether NAC or NACA can effectively inhibit nuclear cataract formation.

The results of this study for the first time reveal that NACA is a promising therapeutic agent for oxidative stress-related ocular diseases, particularly for cataracts. The ability of NACA to effectively reduce lens opacity, preserve lens structure, and modulate key oxidative stress regulators highlights its multifaceted protective mechanisms. Moreover, its safety and efficacy in both ex vivo and in vivo aging-related cataract models underscore its potential for clinical translation. The study relies on mouse models and ex vivo rat lens organ culture, which, while useful for understanding oxidative stress-induced cataractogenesis, may not fully replicate the complex biochemical and structural changes occurring in human cataracts. Future research should focus on exploring the long-term effects of NACA treatment, optimizing its delivery mechanisms, and evaluating its efficacy in clinical trials. Differences in antioxidant defense mechanisms and metabolic rates between rodents and humans should be considered when extrapolating these findings to clinical applications. Additionally, investigating its effects in combination with other antioxidant therapies may provide further insights into its potential as a multitargeted approach for cataract prevention.

## 5. Conclusions

NACA demonstrated significant cytoprotective and antioxidant effects in mitigating oxidative stress-induced damage in LECs and lenses. The ability of NACA to suppress ROS levels, modulate *Txnip* expression, and preserve lens transparency underscores its therapeutic potential. These findings provide a strong foundation for further investigation of NACA as a novel strategy for the prevention and management of cataracts.

## Data Availability

The data presented in this study are available on request from the corresponding author. The data are not publicly available due to institutional guidelines and ethical considerations related to the handling and usage of animal experimental data.

## Supplementary Materials

The following supporting information can be downloaded at: https://www.mdpi.com/article/10.3390/biom15030442/s1, Figure S1: Representative images of lenses from 56-week-old C57BL/6 mice treated with PS (control, N = 4; 4 eyes), or NACA at concentrations of 1, 2, and 5 mM (N = 4; 4 eyes per concentration) for 4 weeks.

## Abbreviations

The following abbreviations are used in this manuscript:

ANOVA	analysis of variance
BSO	L-buthionine-(S,R)-sulfoximine
DMEM	Dulbecco’s modified Eagle’s medium
FBS	fetal bovine serum
GAPDH	glyceraldehyde 3-phosphate dehydrogenase
GSH	glutathione
H2-DCF-DA	dichlorodihydrofluorescein diacetate
H_2_O_2_	hydrogen peroxide
HLEC	human lens epithelial cell
LEC	lens epithelial cell
MLEC	mouse lens epithelial cell
NAC	N-acetylcysteine
NACA	N-acetylcysteine amide
NACET	N-acetyl-L-cysteine ethyl ester
NLRP3	nucleotide-binding oligomerization domain-like receptor family pyrin domain containing 3
PRDX6	peroxiredoxin 6
PS	physiological saline
PSC	posterior subcapsular cataract
ROS	reactive oxygen species
RT-qPCR	real-time reverse transcriptase-quantitative PCR
SD	standard deviation
tBHP	tert-butyl hydroperoxide
TRX	thioredoxin
TXN1	thioredoxin-1
TXNIP	thioredoxin-interacting protein
