# Supplementary material for: Topical Instillation of N-Acetylcysteine and N-Acetylcysteine Amide Impedes Age-Related Lens Opacity in Mice
Source: Biomolecules. 2025 Mar 19;15(3):442. doi: 10.3390/biom15030442 (PMC11940285; doi:10.3390/biom15030442)
Supplement: Supplementary file 1 [file biomolecules-15-00442-s001.zip › biomolecules-3516322-supplementary.pdf]

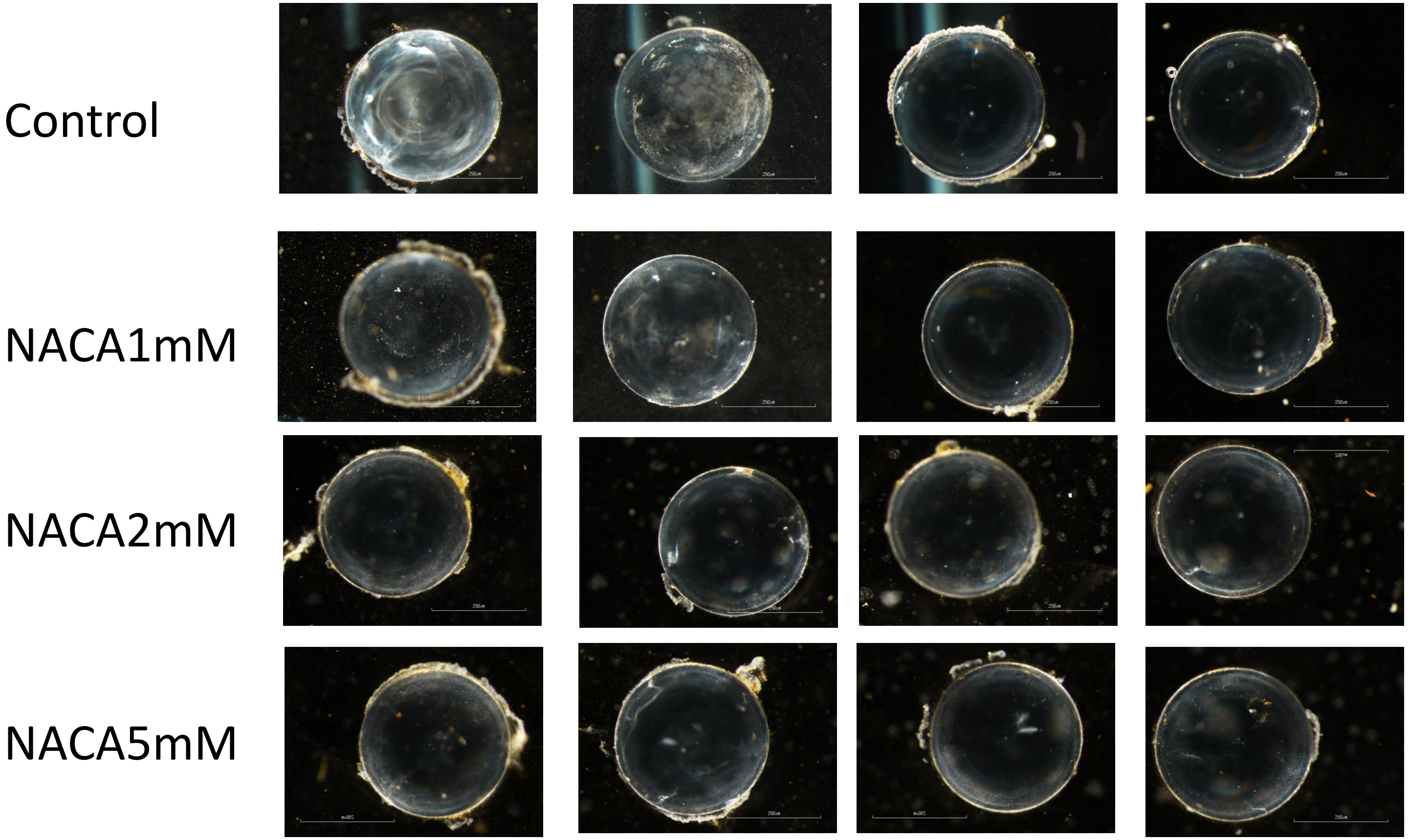

Figure S1: Representative images of lenses from 56-week-old C57BL/6 mice treated with PS (control, N = 4; 4 eyes), or NACA at concentrations of 1, 2, and 5 mM (N = 4; 4 eyes per concentration) for 4 weeks. Lens opacity was assessed under a dark-field stereoscopic microscope. Lenses from the PS-treated group exhibited significant opacity, whereas lenses from the NACA-treated groups demonstrated markedly reduced opacity. Notably, the 2 and 5 mM concentrations of NACA appeared more effective than the 1 mM concentration in suppressing lens opacity, with no noticeable difference between the 2 and 5 mM concentrations. Thus, based on these results, the 2 mM concentration of NACA was selected for further experiments.
